# Potential of Fourier Transform Mass Spectrometry (Orbitrap and Ion Cyclotron Resonance) for Speciation of the Selenium Metabolome in Selenium-Rich Yeast

**DOI:** 10.3389/fchem.2020.612387

**Published:** 2020-12-09

**Authors:** Katarzyna Bierla, Giovanni Chiappetta, Joëlle Vinh, Ryszard Lobinski, Joanna Szpunar

**Affiliations:** ^1^Universite de Pau et des Pays de l'Adour, E2S UPPA, CNRS, IPREM UMR5254, Institut des Sciences Analytiques et de Physico-chimie pour l'Environnement et les Matériaux, Hélioparc, Pau, France; ^2^SMBP, ESPCI Paris, Université PSL, CNRS, Paris, France; ^3^Laboratory of Molecular Dietetics, IM Sechenov First Moscow State Medical University (Sechenov University), Moscow, Russia; ^4^Faculty of Chemistry, Warsaw University of Technology, Warsaw, Poland

**Keywords:** orbitrap, selnometabolomics, FT ICR MS, fourier transfom, selenium speciation

## Abstract

The evolution of the field of element speciation, from the targeted analysis for specific element species toward a global exploratory analysis for the entirety of metal- or metalloid-related compounds present in a biological system (metallomics), requires instrumental techniques with increasing selectivity and sensitivity. The selectivity of hyphenated techniques, combining chromatography, and capillary electrophoresis with element-specific detection (usually inductively coupled plasma mass spectrometry, ICP MS), is often insufficient to discriminate all the species of a given element in a sample. The necessary degree of specificity can be attained by ultrahigh-resolution (R >100,000 in the *m/z* < 1,000 range for a 1 s scan) mass spectrometry based on the Fourier transformation of an image current of the ions moving in an Orbitrap or an ion cyclotron resonance (ICR) cell. The latest developments, allowing the separate detection of two ions differing by a mass of one electron (0.5 mDa) and the measurement of their masses with a sub-ppm accuracy, make it possible to produce comprehensive lists of the element species present in a biological sample. Moreover, the increasing capacities of multistage fragmentation often allow their *de novo* identification. This perspective paper critically discusses the potential state-of-the-art of implementation, and challenges in front of FT (Orbitrap and ICR) MS for a large-scale speciation analysis using, as example, the case of the metabolism of selenium by yeast.

## Introduction

For decades, hyphenated techniques combining the selectivity of chromatography or capillary electrophoresis with an atomic absorption (AAS), fluorescence (AFS), microwave plasma source emission (MIP AES), or inductively coupled plasma mass spectrometry (ICP MS) detectors remained a standard tool for speciation analysis (Lobinski, 1997; Szpunar et al., 2003). Their success was based on the detector's selectivity for the target element and on the baseline separation of the target species from other species of the same element.

The increasing detection sensitivity has put in evidence the fact that, in many cases, chromatography, even multidimensional, was unable to ensure the molecular specificity of the analytical signal measured. When all the complexes of an element with proteins and low-molecular-weight biological ligands have to be determined within one run, the chromatographic peak capacity becomes insufficient to offer the baseline separation of all its compounds of interest; in addition, risks, inherent to chromatography, of incomplete recoveries, and species transformation occur (Lobinski et al., 2010). This has been leading to the shift of paradigm in speciation analysis. Rather than by chromatography, the specificity is more and more often ensured by the separation of ions, characteristic of the individual metal complexes, in a mass spectrometer. Electrospray MS, which has been considered for a long time as a technique complementary to ICP MS and used to confirm or enable the species identification, is becoming a standalone tool for speciation analysis. It can be used in direct (infusion) mode or coupled to HPLC sample introduction and is able to produce data on tens or hundreds of element species in a single mass spectrum.

The use of electrospray MS as a standalone technique for speciation analysis imposes conditions on its performance, especially in terms of resolution and mass accuracy. Not only should the target species ion be separated from the ions of other species of the same element, but also it should be separated from any other ion produced by the ionization of other concomitant molecules. Complex natural mixtures may contain several hundreds of thousands of molecules with close mass differences spread over a wide mass range (Palacio Lozano et al., 2020). The resolution of at least 0.1 mDa is necessary to allow the separation of all the generated ions (Kim et al., 2006). Moreover, the baseline separation of the ions is a *sine qua non*-condition for the sub-ppm mass accuracy, which is important for the confident assignment of individual molecular compositions (empiric formulae) within a mass spectrum.

The demanded figures of merit can only be obtained by MS techniques based on the Fourier transformation of an image current of the ions moving in an Orbitrap or an ion cyclotron resonance (ICR) cell (Marshall and Hendrickson, 2008). The image current is detected as a function of time and is recorded as a composite sum of sinusoidal waves with different frequencies, referred to as a transient. A Fourier transform is applied to this signal to convert it to the *m/z* domain and produce a mass spectrum. Consequently, detailed compositional profiles, with many thousands of unique molecular (empiric formula) assignments, can be obtained from a single mass spectrum. Dedicated data mining procedures have to be used to extract metal speciation-related data from global datasets. Furthermore, multistage fragmentation and the analysis of the dissociation patterns offer data enabling the structural characterization of element species and their unequivocal identification (Dernovics and Lobinski, 2008a).

The emerging paradigm in speciation analysis is therefore based on the assumptions that (i) large numbers of species of metal/metalloids can be ionized in parallel with all the other organic matrix constituents; (ii) all the ions can be ultimately separated in a mass spectrometer; (iii) the molecular masses can be measured with accuracy allowing the unambiguous determination of the empiric formula; (iv) information on element-species of interest can be extracted from large data sets, and (v) these metal species can be structurally characterized by multistage fragmentation and unambiguously identified.

When implemented to a real case study, however, this hypothetical workflow raises a number of practical questions that are discussed below using an example of speciation of selenium following its metabolism by yeast. Such a process leads to hundreds of selenium species of which the identity still remains limited.

## Interest in the Identification of the Products of the Selenium Metabolism by Yeast

Selenium is an essential element for human and animal nutrition (Rayman, 2012). The addition of Se to the diet through supplements or fortified food/feed is increasingly common owing to the often suboptimal content of Se in staple foods in many countries (Fairweather-Tait et al., 2011; Rayman, 2012). The popular basis of such supplements is Se-rich yeast (Fagan et al., 2015) produced by growing different yeast varieties, usually *Saccharomyces cerevisiae* and *Candida utilis*, in the presence of selenite or selenate. Protein-incorporated selenomethionine is the primary selenium species produced in *S. cerevisiae* (Fagan et al., 2015), whereas selenohomolanthione is the most abundant species (ca. 80%) produced by *C. utilis* (Bierla et al., 2017). In either case, in a good-quality yeast, selenium is metabolized completely to organic forms and plethora of LMW (<1,000 Da) species, referred to as selenometabolome, are produced. Its detailed characterization is essential for the control of the reproducibility of the fermentation process, variance of different strains, and traceability of the product's origin (Ward et al., 2019). Furthermore, as some of the metabolites may have superior beneficial activity (and, on the other hand, others can be toxic), the motivation of the producers to offer a well-characterized unique product is evident. Also, it is important for the consumer to understand differences between formulations available on the market.

## Characterization of the Se-Rich Yeast Metabolome: State-of-the-Art

The selenium non-proteic metabolome constitutes between 10% and more than 90% of the total selenium present. It is isolated with quantitative recovery by extraction with water or water-ethanol mixture. The extraction is reproducible within a few percent in terms of retention time of selenium species and their peak intensity is demonstrated by HPLC-ICP MS. The extract is sometimes pre-concentrated by freeze-drying and often fractionated by size exclusion (SEC) and/or anion-exchange chromatography (AEC). The analysis can be carried out either directly (by infusion) or by coupling of HILIC/reversed-phase HPLC to ESI MS. The water-soluble high molecular weight (proteic) species can be eliminated by 3-kDa cutoff filtration before the analysis. Table 1 summarizes the mass spectrometry-based procedures which have contributed to the characterization of the metabolome of the Se-rich yeast. The analyses have been carried out by TOF MS or Orbitrap MS.

**Table 1:** Summary of the mass spectrometry reports of the characterization of the Se-rich yeast metabolome.

**Year**	**Instrument**	**Sample (aqueous** **extract) prior fractionation**	**Resolution**	**Sample introduction (transient)**	**Nr of species**	**Ref**.
2002	QTOF MS (QSTAR2 Pulsar, AB/MDS SCIEX)	SEC/AEC		Infusion: 1 min (60 cycles of 1 s)	5 out of 8 identified (out of 8 detected)	McSheehy et al., 2002
2006	Q-TOF MS (QSTAR XL, Applied Biosystems)	SEC		RP nanoHPLC (0.8 s)	7 identified (5 novel)	García-Reyes et al., 2006
2008	LTQ-Orbitrap MS (Thermo Fisher Scientific)	SEC/AEC	60,000 at *m/z* 400		8 identified (6 novel)	Dernovics and Lobinski, 2008b
2008	LTQ-Orbitrap MS (Thermo Fisher Scientific)	SEC	60,000 at *m/z* 400	HILIC (1 s)	9 identified	Dernovics and Lobinski, 2008a
2009	LTQ-Orbitrap MS (Thermo Fisher Scientificany)	AEC	60,000 at *m/z* 400	HILIC or RP HPLC (1s)	9 detected 8 identified 5 novel	Dernovics et al., 2009
2010	LTQ-Orbitrap MS (Thermo Fisher Scientific)	None^*^	30,000	RP HPLC	10 identified	Rao et al., 2010
2012	LTQ-Orbitrap Velos (Thermo-Fisher Scientific)	None		2D (SEC-RP) HPLC	49 identified 30 novel	Preud'homme et al., 2012
2012	LTQ-Orbitrap Velos (Thermo-Fisher Scientific)	SEC		HILIC or RP HPLC	64 detected 52 identified 9 novel	Arnaudguilhem et al., 2012
2017	TOF MS (Agilent TOF 6210)	SEC		HPLC	103 detected	Gilbert-López et al., 2017
2019	Q-TOF MS (Agilent 6545)	Ultrafiltration		HPLC (1 s)	188 detected 14 identified out of 129 novel	Ward et al., 2019

* *75% ethanol*.

The literature reports show a steady increase in the number of detected and confirmed species with a clear lack of the definitive confirmation of the identity, and thus speciation. Indeed, the progress in terms of the identification of the detected compounds has been largely unsatisfactory. Recent studies (Gilbert-López et al., 2017; Ward et al., 2019) show that only a small fraction of the detected, previously unreported compounds could be identified. This clearly demonstrates the need for analytical methods allowing the large-scale speciation analysis of the Se-rich yeast metabolome. A further tangible progress is unlikely to be possible without the implementation of FT ICR MS into the speciation protocols, the improvement of the multistage fragment analysis, and a re-design of the upstream chromatographic fractionation approaches.

## Ultrahigh-Resolution High Mass Accuracy MS: Resolving Power and Accurate Mass Measurement

The unique species identity assignment can only be attained if the ions of interest can be separated from all those produced by any other molecule present. In a landmark thought experiment, Marshall et al. considered all the possible elemental compositions C_*c*_H_*h*_N_*n*_O_*o*_S_*s*_, where c, h, n, o, and s are the numbers of the respective elements in the molecule structure. They concluded that for even-electron ions (*M*+H)^+^ and (*M*–H)^−^, mass resolution and accuracy of 0.1 mDa are generally sufficient to yield a unique elemental composition for molecules of up to 500 Da, even in the most complex natural mixtures (Kim et al., 2006). Consequently, for a broadband accurate (sub-ppm) mass measurement, the discrimination of the difference of one electron (0.5 mDa) is sufficient for the assignment of a unique empiric formula to an *m/z* 300 molecule (Kim et al., 2006). For the metal speciation analysis, this conclusion is likely to be valid even for higher *m/z* and/or for lower resolution values because (i) not all theoretically possible elemental compositions are chemically possible and (ii) the presence of a heteroelement, often with a characteristic isotopic pattern, is a potent discriminatory factor.

**Figure 1:**
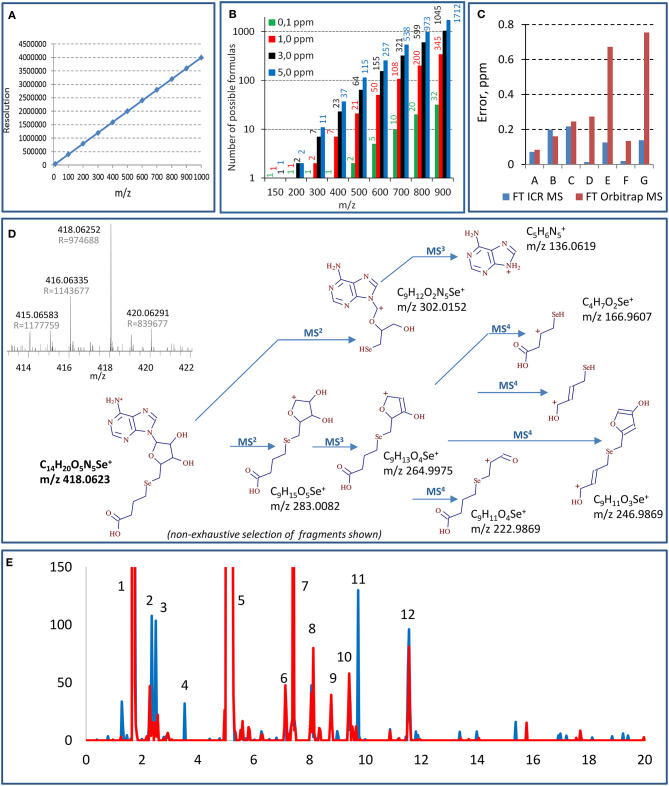
Principal aspects of selenium speciation by ultrahigh-resolution mass spectrometry: **(A)** theoretical resolution necessary for the baseline resolve of equimolar mixtures of species of different *m/z*; **(B)** number of possible molecular formulas for different *m/z* at different levels of mass accuracy (according to Kind and Fiehn, 2006); **(C)** comparison of mass errors observed for different Se-adenosyl derivatives (*m/z* 300–700 Da) in FT Orbitrap MS (Lumos, transient 2.4 s, resolution 1 M) and FT ICR MS (7 T, transient 12.6 s for resolution of 1 M); **(D)** multistage (MS^4^) fragmentation of a previously unreported Se species using QqOrbitrap MS. A high-resolution mass spectrum (FT ICR MS) having allowed the detection of the species an shown in inset; **(E)** example chromatograms generated from the same data set targeting different sets of selenium isotopes (red line—77, 78, and 80, black line—78, 80, and 82). Masses of the selenium species identified in the peaks: 1—348.0199, 431.0569, 449.0674, 453.0387, 487.0144, 471.0492; 2—364.0147; 3—344.0250, 362.0364; 4—416.0459; 5—332.0246, 387.0671, 433.072, 455.0544, 471.0194; 6—404.04677; 7—442.0365; 8—521.0886, 637.0819; 9—475.0830; 10—713.0985, 388.0510, 446.0567, 346.0406; 11—418.0615; 12—681.0100. The compounds are not retention time but mass resolved.

Figure 1A demonstrates that in order to distinguish the species with one electron mass difference, a resolution of 1,400,000 is necessary at *m/z* 400; it increases with the *m/z* of the target molecules. Standard 7T FT ICR MS instruments offer resolution of ca. 750,000 for *m/z* 400 although it can be doubled to reach 1,500,000 (*m/z* 400, 4 s transient signal) (Cho et al., 2017). The highest resolutions reported for *m/z* 400 in real sample measurement conditions, 1,200,000 (3-s transient, magnitude mode) and 2,700,000 (for a 6.3-s transient, absorption mode), were reported using a 21-T FT ICR instrument (Palacio Lozano et al., 2020). The resolutions of the new Orbitrap MS models are comparable with the 7-T FT ICR; the highest reported resolution at *m/z* 400 was 841,000 (absorption mode, 3-s transient) (Schmidt et al., 2018). It is worth noting that these resolutions can be improved by instrumental and/or data processing techniques; the acquisition of full transients for custom-designed signal processing allowed a resolution of 3.5 M at *m/z* 600 (6-s transient) in a model experiment using Orbitrap Lumos (Tsybin et al., 2019). The resolving power decreases when *m/z* increases (linearly for FT ICR and as square root for Orbitrap). It is proportional to the scan duration, which may favor the chromatographic fraction collection and infusion over HPLC sample introduction. The molecular mass determination provides hypotheses on the identity of the detected selenium compound. Indeed, the higher the accuracy, the shorter the list of candidate molecular formulas (Figure 1B) varying from 1 to 2 candidates for low *m/z* (150) and from 32 to 3,447 candidates for higher *m/z* (900) with the respective mass accuracy at 0.1 and 10 ppm (Kind and Fiehn, 2006). The compound's identity has, however, to be verified by the targeted fragmentation of the ion in question, which also provides the structural information on the molecule.

## Identification of Selenium Species by Ultrahigh-Resolution High Mass Accuracy MS in Se-Rich Yeast

### Ionization of Selenospecies: Infusion vs. HPLC MS

Selenium compounds are typically analyzed as protonated [M+H]^+^ species. As discussed above, ultrahigh resolutions can be readily obtained only for several second transients which require sample introduction by infusion. However, in contrast to the examples of complex matrix analysis, such as petroleomics, reports of selenium speciation in the infusion mode are rare, and none of them concerned a raw unprocessed extract (Casiot et al., 1999; McSheehy et al., 2002). The main reason is a big excess of the concomitant matrix species with regard to the selenium analyte concentration and the consequent suppression of the Se-species ionization efficiency which requires the isolation and purification of the analytes. An additional difficulty is the separation of polar selenium species from the matrix salts. Consequently, various chromatographic methods have been coupled to UHR MS, utilizing separation steps to reduce matrix effects for low-ion-yield species. In particular, the high efficiency of HILIC for the studies of polar selenium compounds should be noted (Aureli et al., 2012; Ouerdane et al., 2013).

### FT ICR MS Detection of Se Species

Figure 1C shows that the mass accuracies reached by a 7-T FT ICR obtained for 7 Se-adenosyl derivatives (*m/z* 300–700 Da) are 0.1 ± 0.1 ppm. The mass accuracy errors obtained by Orbitrap MS may be an order of magnitude higher. The inset in Figure 1D shows a mass spectrum obtained for a previously unreported compound at m/z 418.0630 obtained at 1 M resolution and 0.2 ppm mass accuracy (7 T, 2 s transient). This molecular mass corresponds to a unique combination of C, H, S, N, and Se atoms giving the molecular formula of C_14_H_19_O_5_N_5_Se.

### Orbitrap MS^n^ Species Identification

Data-dependent acquisition MS^n^ mode usually fails for selenium speciation unless HPLC is carried out for a sample having undergone multistage purification and enrichment. Concentrations should be high enough to allow the fragmentation even if performed in targeted mode (Ward et al., 2019). Hence, as a rule, MS^2^ (and especially MS^3^ and MS^4^) runs in selenium speciation studies are targeted. In this context, an ion trap instrument with high-accuracy fragment measurement is essential for the structure confirmation and elucidation (Dernovics and Lobinski, 2008a). Figure 1D shows an MS^4^ insight into the structure of a novel selenium compound detected by FT ICR MS. The numerous MS^4^ fragments of various sizes allow one to put forward hypotheses regarding the composition of ions at the M^3^ and M^2^ levels and to validate or reject them as a function of fitting the exact molecular mass. The big number of fragments at the different MS stages, their empiric formula measured with reasonable high accuracy (<3 ppm), and the unique empiric formula of the parent ion obtained owing to the ultrahigh mass accuracy of the FT ICR MS measurement make feasible the unique structure assignment in most cases.

## Auxiliary Tools For Selenium Large-Scale Speciation Analysis

Even if, technically speaking, high-resolution high-accuracy MS detection is sufficient to carry out large-scale metabolomics, some particular features of the system facilitate data analysis and allow the validation of the obtained results.

### Selenium Isotopic Pattern Recognition

As demonstrated above, selenium compounds can be assigned the empiric formula on the basis of a single isotope molecular mass. However, the distinct natural isotopic distribution of selenium is a precious and widely used tool to assist the detection and unambiguous confirmation of the presence of selenium-containing compounds in complex data sets. Selenium has six stable isotopes with detectable distribution: ^74^Se (0.86%), ^76^Se (9.23%), ^77^Se (7.60%), ^78^Se (23.69%), ^80^Se (49.80%), and ^82^Se (8.82%) which can be detected in mass spectra by a unique isotope envelope pattern. The selenium metabolome is also known to contain (about 5 times fewer in number) compounds with two selenium atoms per molecule which requires the search for another characteristic pattern. The pattern to be recognized is distorted by the contribution of minor isotopes of other elements in the molecule (e.g., ^13^C, ^15^N, ^34^S) and suffers from the spectral interferences in the case of insufficient resolution in a complex matrix. A development trend in large-scale selenium speciation is an automatic generation of isotope-pattern selective chromatograms on the basis of a set of parameters such as the number and choice of isotopes, intra-isotope mass defect, and isotope ratio intensity. The definition of the data mining parameters greatly influences the result obtained (Bierla et al., 2018; Gao et al., 2018) as shown for the example chromatograms generated from the same data set using 2, 3, or 5 most abundant selenium isotopes (Figure 1E). Any choice of the parameters carries a certain risk of false negatives or false positives, and a critical evaluation of the generated results is necessary. Also, the detection of the isotopic pattern includes adducts such as [M+Na^+^], [M+K^+^], and [${\text{M+NH}}_{4}^{+}$] that have to be identified (e.g., by their co-occurrence after the chromatographic separation) and eliminated by data treatment (Ward et al., 2019).

### Selenium/Sulfur Homology

A particularity of selenium speciation studies is the fact that selenium shares similar chemical properties with those of sulfur and competes with sulfur in biological processes. As a rule, a selenium species is likely to be accompanied by its sulfur analog which offers multiple opportunities for the analysis of high-resolution high-accuracy MS data sets. As the sulfur metabolic pathways in yeast are known (Mapelli et al., 2012), lists of putative selenium metabolites for targeted analysis can be made. The accurate mass difference measurement allows the identification of the S–Se homologs in data sets. Also, as demonstrated elsewhere (Casiot et al., 1999) the easier availability of sulfur standards allows the validation of the identification of their selenium analogs.

### Lessons From the Chemistry

The observed multitude of detected selenium species is likely to come from the unspecific oxidation and the formation of Se–Se and/or Se–S bonds. The moment in which the oxidation occurs (production process, storage, extraction) is unknown. The acquisition of a chromatogram in the reducing conditions offers direct access to the identity of the–SeH containing original species and facilitates the interpretation of data.

## Outlook

Ultrahigh resolution mass spectrometry is likely to be the ultimate tool for speciation analysis as it inherently offers the required species specificity in complex matrices as demonstrated by recent studies of metal porphyrins in crude oils (Palacio Lozano et al., 2020) or metallophores in the environment (Boiteau et al., 2019). The instrumental power of state-of-the-art FT instruments (ICR and Orbitrap) seems to be sufficient for large-scale selenium speciation in a biological matrix, especially that the Se isotopic pattern can be exploited to improve data mining. However, the potential of FT ICR MS, and especially HPLC-FT ICR MS for the detection of Se species, is still largely unexplored. MS coupled to prior separation is required to differentiate structural isomers of species with the same elemental composition whereas ion mobility MS is expected to provide meaningful complementary data regarding the presence of conformers. Whereas, the large-scale mapping of the entire yeast metabolome is within the reach of high-resolution high-mass-accuracy MS, the quantification of all the individual species by this approach seems to be a remote goal. Its achievement requires the availability of individual standards of the Se species to be quantified.

## Data Availability Statement

The datasets presented in this study can be found in online repositories. The names of the repository/repositories and accession number(s) can be found at: EBI, MTBLS2132.

## Author Contributions

KB, GC, and JV carried out the expeiments. KB, JS, and RL planned the work. JS and RL prepared the draft. All authors completed and appoved the manuscript.

## Conflict of Interest

The authors declare that the research was conducted in the absence of any commercial or financial relationships that could be construed as a potential conflict of interest.
